# Supplementary material for: Metabolic and mitochondrial dysregulation in CD4+ T cells from HIV-positive women on combination anti-retroviral therapy
Source: PLoS One. 2023 Oct 10;18(10):e0286436. doi: 10.1371/journal.pone.0286436 (PMC10564234; doi:10.1371/journal.pone.0286436)
Supplement: S1 Fig — (DOCX) [file pone.0286436.s001.docx]

SSC- A

Lymphocytes 39.8% Singlets FSC 99.7% Singlets SSC 99.8% Viable cells 92.7%


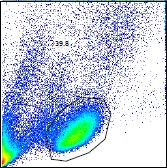

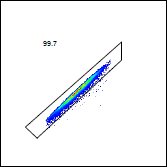


FSC-A


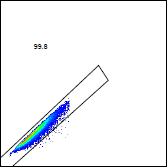

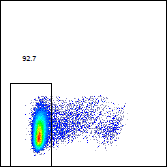

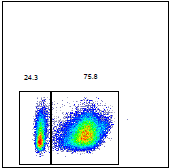

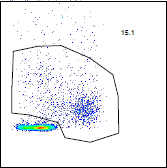

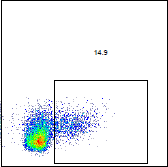


FSC-

H

SSC-

H

SSC-

A

FSC-A

SSC-A

Fixable viability

Stain 780

Dendritic cells

14.9%

CD4^+^ cells

CD3- cells

24.3%

CD3^+^ cells

75.8%

PeCy7 CD11c

Natural killer

PerCP Cy5.5 CD3

BV421 CD8

Cells 15.1%


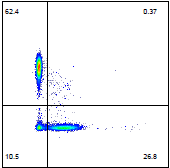


62.4%

CD8^+^ cells

26.8%

SSC-A

SSC-A

PeCF 594 CD4

AF700 CD16

BV510 CD56

**S1 Fig.** Gating strategy for the different leukocyte populations

1
