# Supplementary figures and images for: Metabolic and mitochondrial dysregulation in CD4+ T cells from HIV-positive women on combination anti-retroviral therapy
Source: PLoS One. 2023 Oct 10;18(10):e0286436. doi: 10.1371/journal.pone.0286436 (PMC10564234; doi:10.1371/journal.pone.0286436)

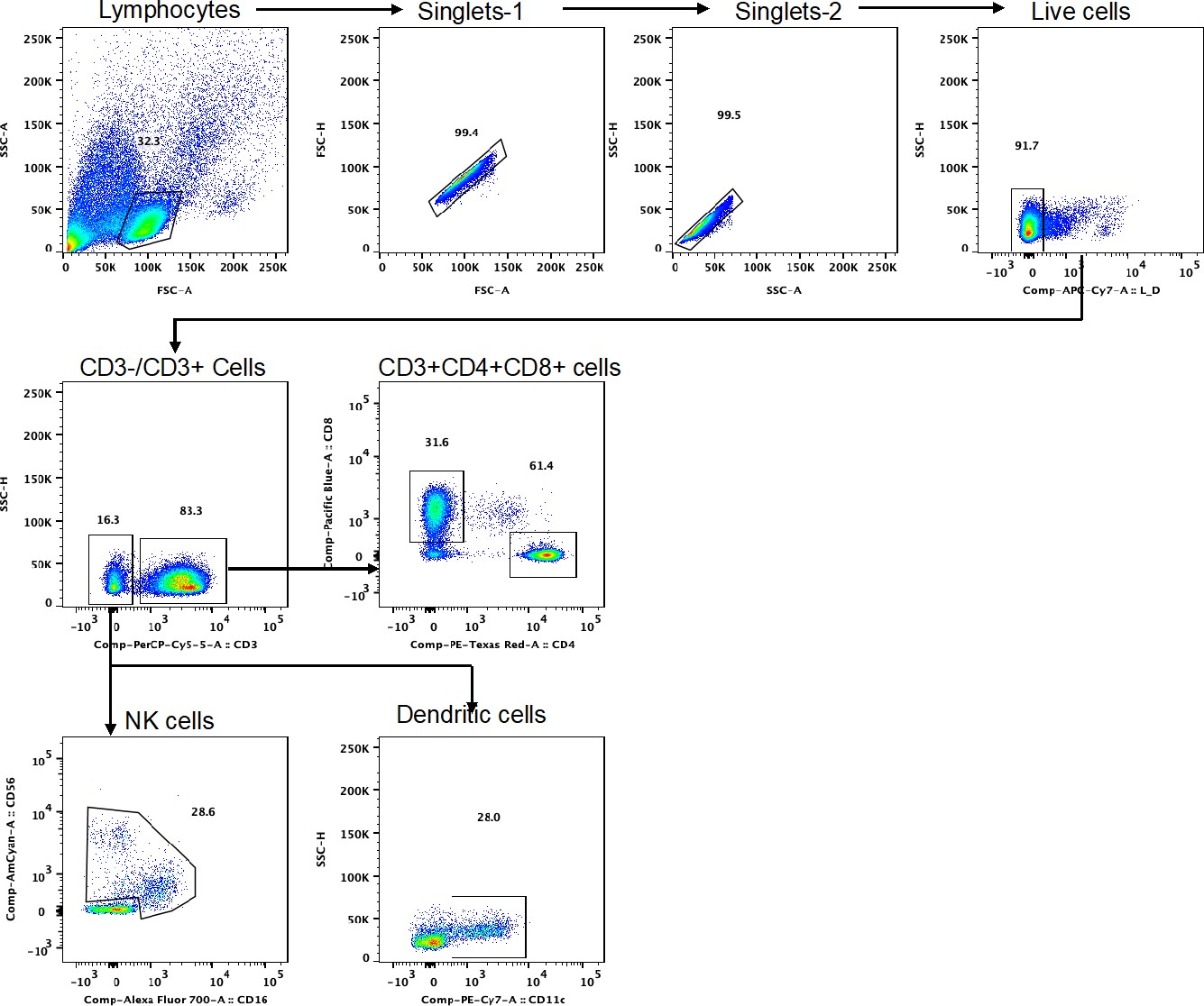


**S2 Fig.** Gating strategy for the different leukocyte populations

2

Supplement: S2 Fig — (DOCX) [file pone.0286436.s002.docx]

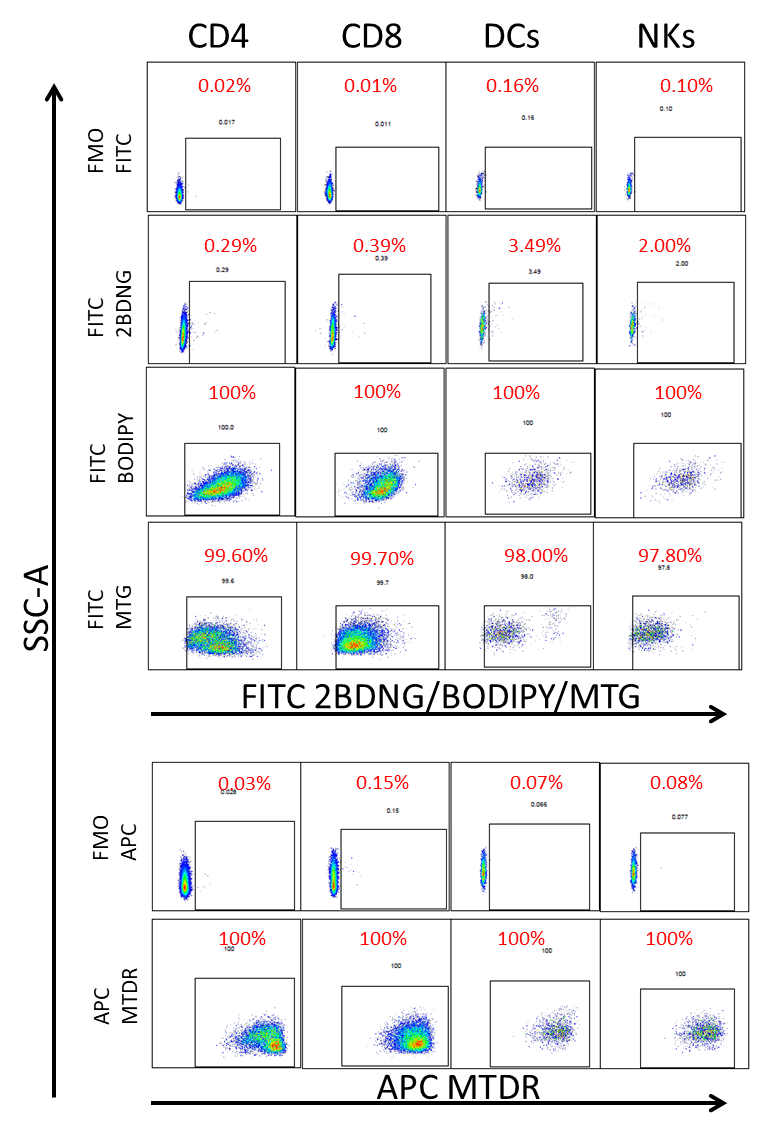


**S3 Fig.** Gating strategy for the different substrate uptake by the different leukocytes

3

Supplement: S3 Fig — (DOCX) [file pone.0286436.s003.docx]
