# Supplementary material for: Metabolic and mitochondrial dysregulation in CD4+ T cells from HIV-positive women on combination anti-retroviral therapy
Source: PLoS One. 2023 Oct 10;18(10):e0286436. doi: 10.1371/journal.pone.0286436 (PMC10564234; doi:10.1371/journal.pone.0286436)
Supplement: S1 Table — (DOCX) [file pone.0286436.s004.docx]

| **Patient ID#** | **Opportunistic infections prevention** | **ART regimen** | **Time on ART (months)** | **CD4**  **counts** | **Viral loads** |
| --- | --- | --- | --- | --- | --- |
| L015-0222005 | COTRIMOXAZOLE | LAMIVUDINE/NEVIRAPINE/TENOFORVIR | 125 | 308 | Undetected |
| L015-0222006 | SEPTRIN | LAMIVUDINE/EFAVIRENZ/ZIDOVUDINE | 104 | 88 | 50 |
| L015-0222007 | SEPTRIN | LAMIVUDINE/NEVIRAPINE/ZIDOVUDINE | 125 | 839 | Undetected |
| L015-0222008 |  | LAMIVUDINE/NEVIRAPINE/TENOFORVIR | 115 | 560 | Undetected |
| L015-0222009 | COTRIMOXAZOLE | EFAVIRENZ/LAMIVUDINE/TENOFORVIR | 38 | 478 | 420 |
| L015-0222011 |  | EFAVIRENZ/LAMIVUDINE/TENOFORVIR | 108 | 315 | Undetected |
| L015-0222010 | COTRIMOXAZOLE | EFAVIRENZ/LAMIVUDINE/TENOFORVIR | 75 | 519 | Undetected |
| L015-0222013 | COTRIMOXAZOLE | EFAVIRENZ/EMTRICITABINE/TENOFORVIR | 120 | 1460 |  |
| L015-0222014 | COTRIMOXAZOLE | EFAVIRENZ/LAMIVUDINE/TENOFORVIR | 150 | 1458 | Undetected |
| L015-0222015 | SEPTRIN | EFAVIRENZ/LAMIVUDINE/TENOFORVIR | 58 | 500 | Undetected |
| L015-0222016 | COTRIMOXAZOLE | LAMIVUDINE/NEVIRAPINE/TENOFORVIR | 100 | 286 | 600 |
| L015-0222012 |  |  |  | 146 | 142360 |
| L015-0222017 | COTRIMOXAZOLE | EFAVIRENZ/LAMIVUDINE/TENOFORVIR | 50 | 764 | Undetected |
| L015-0222018 | COTRIMOXAZOLE | LAMIVUDINE/NEVIRAPINE/ZIDOVUDINE | 108 | 772 | Undetected |
| L015-0222019 | SEPTRIN | LAMIVUDINE/NEVIRAPINE/ZIDOVUDINE | 95 | 529 | Undetected |
| L015-0222020 |  | EFAVIRENZ/LAMIVUDINE/TENOFORVIR | 112 | 1175 | Undetected |

**S1 Table.** Clinical information of HIV-1 infected individuals on ART treatment

4
